# Renal Medullary Carcinoma is a Diagnosis Worth Considering: Case Report

**DOI:** 10.4021/wjon222w

**Published:** 2010-08-29

**Authors:** Christopher Thomas, Azhar Khan, Hari Khrishnan, Gary Das

**Affiliations:** a Department of Urology, Mayday University Hospital, Croydon, London, UK

**Keywords:** Medullary cell cancer, sickle cell

## Abstract

Medullary Renal Carcinoma is a rare, highly malignant neoplasm that originates in the renal medulla and typically affects young black patients with sickle cell trait. We report the case of a 17-year-old boy with a symptomatic left renal tumour. CT revealed that the mass originated from the kidney and was associated with a large para-aortic lymph node mass. Hemoglobin electrophoresis showed sickle cell trait and a needle biopsy confirmed the diagnosis of Renal Medullary Carcinoma. We discuss the obscurity and implications of such a diagnosis. It is essential that clinicians are aware of this diagnosis as any delay can be fatal in the outcome of this highly aggressive and extremely rare cancer.

## Introduction

Renal medullary carcinoma (RMC) is a devastating but rare renal tumour that most commonly affects young black patients with sickle cell trait. It is a highly aggressive and infiltrative malignancy with an almost universally poor outcome as most of the patients present with metastatic disease at diagnosis. We herein describe the case of a medullary renal cell carcinoma that highlights the importance of a high index of suspicion in black patients with hematuria and/or flank pain, and the need to perform hemoglobin electrophoresis and renal phase CT as an early minimum standard of investigation.

## Case Report

A 17-year-old Afro-Caribbean boy presented with a one year history of intermittent left loin pain, hematuria and fever. He had been previously treated in the community for recurrent urinary tract infections. There was no history of nausea, vomiting or weight loss. On examination, the abdomen was soft but tender in the left loin region. No masses were palpable within the abdomen at presentation. A full blood count and renal function screen was normal, while urine analysis only showed proteinuria. Renal tract ultrasound indicated a 50 mm x 25 mm avascular area which was echogenic and raised the possibility of pyelonephritis complicated with a renal abscess. Patient was initially treated with intravenous antibiotics but failed to respond.

**Figure 1:**
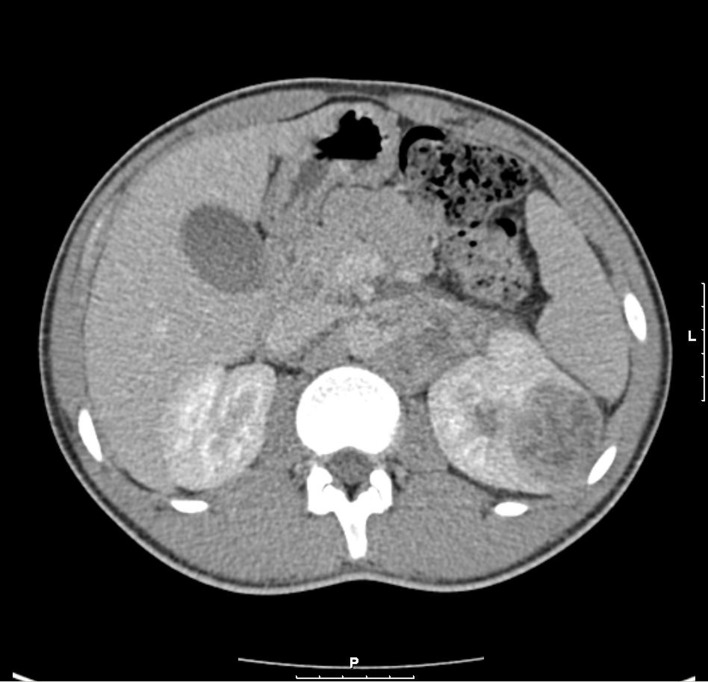
CT shows left renal enhancing lesion with para-aortic lymph node mass.

**Figure 2:**
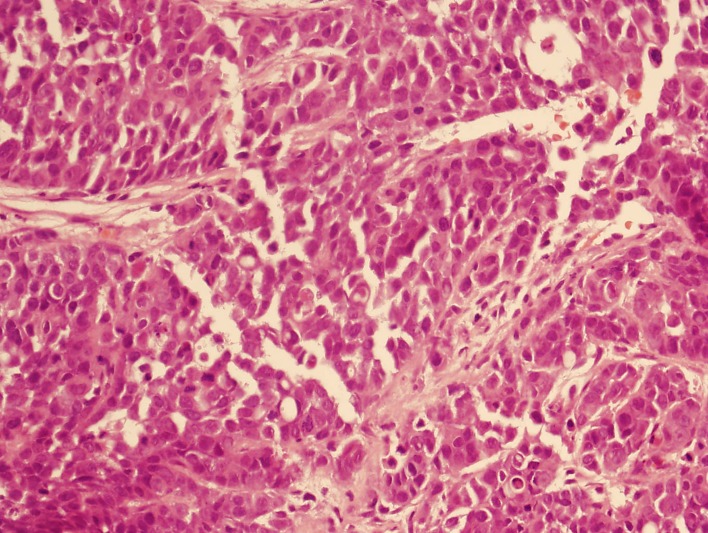
Solid sheet of cells with eosinophilic cytoplasm and prominent nucleoli.

A 3-phase CT abdomen showed the presence of an enhancing mass lesion expanding the superolateral aspect of the left kidney with an extensive abnormal para-aortic lymph nodes mass encasing the renal vessels (Fig. 1). A biopsy of the left kidney mass showed renal parenchyma widely infiltrated by a poorly differentiated adenocarcinoma showing both solid sheets of cells, trabecular arrangements and a micro glandular architecture. The cells showed marked cytological pleomorphism and frequent mitoses with focal necrosis. Lymphovascular invasion was also seen. Interestingly, a red blood cell (RBC) visible in one of the slide looked sickled and raised the possibility of sickle cell disease (Fig. 2). Subsequently, a sickle cell screen was instructive of carrier status with electrophoresis showing HbA 51.0% and HbS 37.5%. On further immunohistology, the end immunoprofile (cytokeratin positive, vimentin positive and CK5, 6 negative) was in keeping with a diagnosis of medullary carcinoma of kidney.

Radical open nephrectomy through thoraco-abdominal approach was performed together with partial clearance of para-aortic lymph node mass as it was matted and fixed to the aorta. Per operatively, invasion was also noted into the psoas muscle and colonic mesentery. After postoperative recovery, patient was given six cycles of gemcitabine and cisplatin, and showed a good partial response with good volume reduction of retroperitoneal/para-aortic nodal disease.

## Discussion

In 1995, Davis *et al* described renal medullary carcinoma (RMC) as a new tumour classification, suggesting a distinct relationship between chronic hypoxia and tumour development [1]. The environment of the renal medulla is characterized by acidosis and hypoxia promoting hemoglobin S polymerization and red blood cell sickling, thereby making this area of the kidney particularly susceptible to changes in oxygen delivery in patients with both sickle cell trait and disease [2].

RMC occurs much more frequently in young males with sickle cell trait than in those with homozygous sickle cell [3]. Early mortality is the rule, with few reports of survival beyond a few months [4]. It typically arises from the renal medulla, and radiologically, the lesions usually show highly aggressive, infiltrating and poorly defined masses with increased echogenicity or enhancement with associated retroperitoneal adenopathy [5]. CT or MRI can be used for diagnosis but a fine-needle aspiration biopsy is recommended because this has a high sensitivity and will show the typical features of RMC.

It is obviously important to differentiate between RMC and other renal tumours. Unlike renal cell carcinoma, most patients with RMC present with hematuria, flank pain and/or a palpable mass. The primary differential diagnosis for RMC is collecting duct carcinoma (CDC) because they do share some features. However, Swatz *et al* [6] described genetic, clinical and histopathological differences between the two, and Srigley and Eble [7] suggested collecting duct carcinoma as most likely to present in a patient without sickle cell trait, primarily in males and later in life (median age of 53 years). Moreover, RMC has predominantly a reticular pattern, whereas CDC more commonly displays a tubular or tubulopapillary patterns and immunohistochemically, CDC typically is cytokeratin 34-E12 and ulex europeus agglutinin 1 lectin positive.

The outcome of renal medullary carcinoma is generally very poor due to the aggressive nature of this neoplasm, late diagnosis and also to its resistance to conventional chemotherapy. The mean survival is about 3 months [1]. The long duration of symptoms in most patients perhaps offers the best hope for future improvements in survival rate mainly with early diagnosis, as there is currently little literature to suggest any true success with surgical, chemo- and/or radio-therapeutic modalities. It is essential that primary healthcare doctors and specialist urologists are aware of this diagnosis so that early investigations can be arranged in all young black patients with sickle cell trait presenting with hematuria and/or loin pain.
